# Fermentation by Probiotic *Lactobacillus gasseri* Strains Enhances the Carotenoid and Fibre Contents of Carrot Juice

**DOI:** 10.3390/foods9121803

**Published:** 2020-12-04

**Authors:** Yue Xu, Mya Myintzu Hlaing, Olga Glagovskaia, Mary Ann Augustin, Netsanet Shiferaw Terefe

**Affiliations:** CSIRO Agriculture and Food, 671 Sneydes Road, Werribee, VIC 3030, Australia; xuyue930@163.com (Y.X.); Myintzu.hlaing@csiro.au (M.M.H.); olga.glagovskaia@csiro.au (O.G.); maryann.augustin@csiro.au (M.A.A.)

**Keywords:** fructosyltransferases, *Lactobacillus gasseri* DSM 20604, *Lactobacillus gasseri* DSM 20077, reduced calorie, carrot juice, polysaccharides

## Abstract

Carrot juice (straight, 8.5 Brix and concentrated, 15.2 Brix) was fermented by lactic acid bacteria (*Lactobacillus gasseri* strain DSM 20604 or DSM 20077). Fermentation enhanced the nutritional profile of carrot juice. There was a greater sugar reduction (27%) in fermented straight carrot juices than in the fermented concentrated juices (15%). The sugar reduction was independent of the strain used for fermentation. The two *L. gasseri* strains synthesised fructosyltransferase enzymes during fermentation of carrot juice samples that enabled conversion of simple sugars primarily into polysaccharides. The level of conversion to polysaccharides was dependent on the *L. gasseri* strain and juice concentration. Fermentation of carrot juice by *L. gasseri* enables the production of a nutritionally-enhanced beverage with reduced calorie and prebiotic potential. An additional benefit is the increased carotenoid content observed in straight and concentrated juices fermented by *Lactobacillus gasseri* DSM 20077 and the concentrated juice fermented by *Lactobacillus gasseri* DSM 20604.

## 1. Introduction

The rise of vegetarianism and prevalence of lactose intolerance has driven consumer’ demand for functional non-dairy products [1,2]. Fermentation enables the production of functional fruit and vegetable juices as an alternative to fermented dairy beverages, with potential prebiotic and probiotic benefits. Probiotic organisms have the capacity to grow and survive in plant substrates, which is influenced by the inherent characteristics of the plant species [1] and the metabolic activity of the organisms. During fermentation, the components of the original food matrix are converted into a range of new metabolites by the activity of endogenous and microbial enzymes. The concentration of many bioactive compounds which present at low concentrations in the unfermented material are increased during fermentation and new bioactives may be formed as a result of the action of the microorganisms [3]. Furthermore, fermentation of fruits and vegetables improve the bioavailability of micronutrients such as iron [4] and vitamin C [5] and phytochemicals such as β-carotene, betaine [5] and the bioaccessibility, bioavailability and bioactivity of polyphenols and glucosinolates [6,7,8]. Transformation of plant substrates during fermentation also yields bioactive peptides, short chain fatty acids and polysaccharides while reducing anti-nutritional compounds [3,9]. Some beneficial bioactive molecules that are increased after fermentation of vegetables possess antioxidative and anti-inflammatory effects [10,11].

Recently, *Lactobacillus gasseri* strains have attracted attention due to their probiotic properties [12,13,14,15] and their ability to produce fructosyltransferase enzymes that catalyze the conversion of sucrose into prebiotic fructans [16,17,18]. Genomic and experimental evidences indicate that *L. gasseri* strains possess probiotic traits including tolerance to low pH, resistance to bile salts, adhesion to host epithelium and production of antimicrobial bacteriocins with inhibitory activity against pathogens [18,19]. Moreover, human clinical trials have shown that *L. gasseri* strains help to maintain vaginal homeostasis and mitigate diarrhea and helicobacter pylori infection [19]. Recent animal model studies have shown that the administration of *L. gasseri* improves glucose tolerance and reduces weight gain in rats [13], reduces hepatic toxicity induced by colorectal cancer in mice [12], and prevents sepsis in *Pseudomonas aeruginosa* infected wounds in murine models [14]. In addition to their probiotic effect, *L. gasseri* are known to produce prebiotic compounds. For instance, *L. gasseri* DSM 20604 synthesise the well-known prebiotic fibre inulin and inulin type fructo-oligosaccharides as well as levan whereas *L. gasseri* DSM 20077 synthesise levan [18], which have been shown to have prebiotic benefits through in vitro studies [20] and better bifidogenic effects than inulin [21]. Anwar et al. [18] investigated the synthesis of fructans by three *L. gasseri* strains viz. DSM 20604, DSM 20077 and DSM 20243. They observed that DSM 20604 synthesises both inulin and levan while DSM 20077 synthesise levan only when cultured in De Man, Rogosa, Sharpe (MRS) media with the glucose replaced by sucrose. DSM 20604 did not only produce inulin but also fructo-oligosaccharides with degree of polymerization (DP) from 2 to 13. On the other hand, DSM 20243 was not capable of synthesizing fructans although it possesses the gene encoding fructosyltransferases, which was attributed to a premature termination of this gene by a stop codon [18]. Another study showed that the optimum condition for the biosynthesis of inulin from sucrose by the recombinant inulosucrase from *L. gasseri* DSM 20604 was pH 5.5 and 25 °C at an enzyme dosage of 0.5 U/g sucrose. Inulin production increased with increase in sucrose concentration and the highest inulin yield at 50% sucrose concentration and under optimal pH and temperature was achieved after 1.5 h of enzymatic reaction. Further increase in reaction time resulted in lower yield which was attributed to possible inulin decomposition [16]. *L. gasseri* fructosyltransferases can also be used for the synthesis of raffinosyl-oligosaccharides. Diez-Municio [22] used inulosucrase from *L. gasseri* DSM 20604 for the synthesis of raffinosyl-oligosaccharides from raffinose with DP of 4 to 8. The maximum yield was 33.4% with respect to the weight of raffinose at raffinose concentration of 50 % (*w*/*v*) [22].

The objective of this study was to evaluate the feasibility of producing a synbiotic plant-based beverage via fermentation using probiotic *L. gasseri* cultures as starters. We hypothesised that fermentation of a sugar rich plant substrate with probiotic *L. gasseri* strains (DSM 20604 and DSM 20077) enables the production of a prebiotic product with reduced sugar content via conversion of some of the simple sugars into prebiotic fructans. Carrot was selected as a substrate since (1) it is one of the most commonly produced and consumed vegetables worldwide (2) it is a low acid vegetable relatively rich in sugars and hence a suitable substrate for growth and metabolic activity of *L. gasseri*, and (3) it is rich in vitamins, minerals, and phytonutrients such as carotenoids and polyphenols. The predominant carotenoid in carrot, β-carotene, has several health benefits including pro-vitamin A activity and preventative effects against cataract and cardiovascular diseases whereas lutein helps in the prevention of macular degeneration [23]. In this study, we evaluated the feasibility of fermenting carrot juice by the two *L. gasseri* stains (DSM 20604 or DSM 20077) and the impact on selected nutritional attributes of carrot juice upon fermentation.

## 2. Materials and Methods

### 2.1. Materials

Fresh carrots were purchased from a local supplier (Coles supermarket, Werribee, VIC, Australia). The two *Lactobacillus gasseri* strains (DSM 20604 and DSM 20077) cultures were obtained from DSMZ (Braunschweig, Niedersachsen, Germany). De Man, Rogosa, and Sharpe media (MRS broth, CM0359, and MRS agar, CM0359 from Oxoid (Melbourne, Victoria, Australia) and Maximum Recovery Diluent (MRD) were purchased from Thermo Fisher Australia (Scoresby, Victoria, Australia). All the other chemical and biochemical reagents were analytical or High performance liquid chromatography (HPLC) grade and purchased from Merck (Kilsyth, VIC, Australia) or Sigma-Aldrich (Castle Hill, NSW, Australia).

### 2.2. Carrot Juice Preparation

The carrots were washed, steamed at 100 °C to a core temperature of 80 °C, cooled down with ice, and then blot dried prior to juicing. The juicing process included shredding and compression using cold press juicer (Fresh Press, Model FP100, Laverton, Victoria, Australia). The carrot pre-heating conditions were established after preliminary trials at different conditions to enable the inactivation of pectin methylesterase so as to prevent pectin precipitation and sedimentation [24]. The carrot juice (~8.5 Brix) was concentrated (15 °Brix, ~2-fold) using a forward osmosis membrane system (Porifera forward osmosis laboratory system, San Leandro, CA, USA). The concentrated juice was then pasteurised at 100 °C for 15 s using a laboratory scale ultra hight temperature (UHT) processing system (Armfield, DKSH, Zurich, Switzerland). The concentrated juice was used in all the experiments as is (concentrated juice) or was diluted with sterilised Milli-Q water (1:1 *v*/*v* dilution) to obtain straight juice samples.

### 2.3. Preparation of Starter Cultures

The pellets of *L. gasseri* DSM 20604 and *L. gasseri* DSM 20077 cultures (as obtained from DSMZ) were inoculated into a 10 mL MRS broth and were incubated at 37 °C for 48 h under anaerobic conditions to produce the primary culture. The secondary culture was prepared by inoculating 10 µL of the primary culture into 30 mL of MRS broth and incubation for 18 h at 37 °C under anaerobic conditions. The cultures were centrifuged at 5000× *g* for 10 min at 17 °C (Sigma 6–16K benchtop centrifuge, Sigma-Aldrich, Castle Hill, NSW, Australia), and were resuspended in 3 mL of MRS broth to yield a concentration of ~10^9^ CFU/mL, which was confirmed by plating the cultures after serial dilution using MRD on MRS agar and incubating at 37 °C for 48 h under anaerobic condition. All of the culture tubes were combined to make one stock solution and 15% glycerol was added to the mixture. The combined cultures were dispensed into 1 mL aliquots and kept frozen at −80 °C until use. On the day of fermentation, the 1 mL culture tubes were removed from the freezer and defrosted in water maintained at 35 °C for 5 min, centrifuged at 5000× *g* for 10 min, and the supernatant was discarded and the pellets were washed twice by resuspending in sterile phosphate buffered saline (PBS) followed by centrifugation. The pellets were finally resuspended in 1 mL sterile milliQ water to obtain ~10^9^ CFU/mL immediately before inoculation into the substrate to be fermented. Each 1 mL culture tube was used for fermenting 200 mL of carrot juice or juice concentrate.

### 2.4. Carrot Juice Fermentation Experiments

The fermentation conditions (i.e., optimal pH of the juice, inoculum size of the starter culture, the need for extra protein source and fermentation temperature) was established based on the results of preliminary experiments conducted at different conditions. Accordingly, each carrot juice sample (200 mL of straight or concentrated juice) was inoculated with the prepared starter culture to a final concentration of 7 log CFU/mL sample. The fermentation experiment was carried out at 30 °C in a shaking water bath at 100 rpm for 24 h. The pH of the carrot juice (~6.0) was not adjusted since it was close to the optimal pH for the growth of the two *L. gasseri* strains (pH 5.5) which was found to be suitable for the growth of the two strains without the addition of an extra protein source or pH adjustment. All fermented samples were kept at 4 °C until further analyses. The fermentation experiments were conducted in triplicate.

### 2.5. Microbial Analysis

The lactic acid count of the carrot juice samples immediately after inoculation and after the end of fermentation were analysed. Samples were diluted using MRD, plated on MRS agar and were incubated at 37 °C for 48 h under anerobic condition.

### 2.6. Analysis of Sugars by HPLC

The concentration of fructose, glucose and sucrose in carrot juices was determined by using High Performance Liquid Chromatography system (HPLC) equipped with Waters Alliance 2690 Separations Module (Waters Inc., Rydalmere, NSW, Australia) and Waters Refractive Index Detector 410 (Waters Inc., Rydalmere, NSW, Australia). Prior to sugar analysis carrot juice samples were mixed with 80% ethanol in the ratio 1 to 4, and centrifuged at 20,000× *g*, 20 °C for 10 min using Eppendorf temperature-controlled centrifuge (Eppendorf, Macquarie park, NSW, Australia). The isocratic separation of sugars in the supernatants was performed on Shodex Asahipak NH2P-50 4E column (4.6 × 250 mm) (Phenomenex Australia, Lane Cove, NSW, Australia) fitted with guard column Shodex Asahipak NH2P-50G (4.6 × 50 mm) (Phenomenex Australia, Lane Cove, NSW, Australia). The column temperature was maintained at 30 °C during the operation. The mobile phase (68% acetonitrile) was isocratically delivered at flow rate of 1 mL/min. The total run time was 15 min. The quantification of the sugars was based on the calibration curves obtained for each sugar, after injecting known concentrations of a standard.

### 2.7. Determination of pH and Titratable Acidity

The pH of the carrot juices during fermentation was monitored and recorded using a pH data logger (4-Channel Data Logger, Version 2.2.3, Ser. No 1230500, EAI instruments, Wembley, Middlesex, UK). The titratable acidity of the samples was measured using an automatic titrator (Titralab 854 titration manager, Radiometric Analytical, Lyon, France) in accordance with the Organisation for economic co-operation and development (OECD) method of fruit juice analysis. The titratable acidity (TA) is expressed as percentage lactic acid equivalent (*v*/*v*).

### 2.8. Extraction and Assay of Fructosyltransferase Enzymes

The extraction and assay of fructosyltransferases in the fermented carrot juice samples were conducted in accordance with the methods of da Silva et al. [25]. Briefly, the fermented juice sample was centrifuged at 5500 rpm for 15 min at 16 °C to remove the cells. This was followed by ammonium sulphate precipitation and centrifugation to recover the crude enzyme extract. The precipitate was re-dissolved in 1 mL of 20 mM sodium acetate buffer (pH 5.2) and was used as crude enzyme extract for assaying fructosyltransferase activity in each sample. The inulosucrase and the levansucrase activities respectively in samples fermented by DSM 20604 and DSM 20077 were assayed using sucrose as a substrate. The substrate solution (10% *w*/*v*) was prepared in 20 mM sodium acetate buffer (pH 5.2) containing 0.05 mg/mL CaCl_2_. The reaction mixture containing the enzyme extract (200 μL) and 800 μL of the sucrose substrate solution was incubated for 10 min at 30 °C. The reaction was stopped by heating the mixture for 5 min at 100 °C to inactivate the enzyme followed by cooling in ice-water. The sample was then mixed with three volumes of 80% (*v*/*v*) ethanol and centrifuged at 25,000× *g* for 10 min. The supernatant was filtered using 0.2 μm pore size syringe filter and the sucrose, fructose and glucose contents were analysed by HPLC as described in Section 2.6. The amount of glucose released represent total enzyme activity whereas the amount of fructose released represent total hydrolytic activity. The transglycosylation activity was calculated as the difference between the total activity and the hydrolytic activity [18]. One unit of total enzyme activity was defined as the amount of enzyme required to release one µmol of glucose or fructose per minute under the assay condition.

### 2.9. Fourier Transform Infrared (FTIR) and Raman Spectroscopy Analysis

All unfermented straight and concentrated carrot juice samples and the samples fermented by the two *L. gasseri* strains (DSM 20604 and DSM 20077) were analysed by FTIR and Raman spectroscopy. The spectroscopic analyses were conducted on the deproteinised crude polysaccharides extracted from the samples after drying with SpeedVac concentrator (Savant™ SC250EXP, Thermo Fisher Australia, Scoresby, Victoria, Australia) at room temperature and 0.5 torr vacuum pressure. The deproteinisation of the samples using the CaCl_2_ method and the extraction of the crude polysaccharides was carried out in accordance with the methods of Huang et al. [26]. The weight of the crude polysaccharide extracts was used as an estimate for the total amount of polysaccharides in the samples.

FTIR spectra were recorded using a FTIR-8400S system (Shimadzu) equipped with a ZnSe attenuated total reflectance prism and a temperature controlled high sensitivity detector (DLATGS detector) (Shimatzu, Kyoto, Japan). The spectra were collected in the wavenumber range of 600 cm^−1^ to 4000 cm^−1^ with a resolution of 4 cm^−1^ for 80 scans. Raman spectroscopy measurements were recorded using a Renishaw InVia confocal Raman spectroscopy equipped with a Leica microscope plus a deep depletion charge-coupled device detector. The incident laser power ~25 mW from 785 nm radiation from diode laser was used for acquiring the spectra from each sample. Each measurement was performed over the selected area of the sample focused under a 20× microscope objective (NA = 0.4 in air). Raman spectra were collected in the 500 to 2000 cm^−1^ range that covers the fingerprint region of most biological materials [27].

Commercially available software (Matlab and OriginPro) were used for data processing. All FTIR and Raman signal processing for multivariate statistical method of principal component analysis (PCA) was performed as previously described [28,29]. The scores plots for the first and second principal components and the corresponding loadings plots were further analysed to extract chemical information and to analyse macromolecular changes in the fermented carrot juice samples.

### 2.10. Data Analysis

All experiments were conducted in triplicate and reported as means ± standard deviation.

A one-way analysis of variance (ANOVA) was applied to evaluate the significance of the difference between mean comparisons of each sample group (*p* value < 0.05). Microsoft excel and Originpro 2020 software (Origin lab corporation, Northampton, MA, USA) were used for statistical analysis.

## 3. Results and Discussion

### 3.1. Production of Fructosyltransferase Enzymes in Fermented Carrot Puree

Following the fermentation of straight and concentrated carrot juices by the two strains, the activity of fructosyltransferase enzymes in the DSM20604 and DSM 20077 fermented samples respectively were examined with sucrose as substrate. Data are presented in Table 1. A significant fructosyltransferase activity was observed both in the straight and concentrated carrot juice samples after fermentation. The total activity was higher in the fermented straight juices compared to the concentrated juice samples, although the difference was not statistically significant (*p* > 0.05) due to substantial sample to sample variability. The hydrolytic activities were similar in all cases, whereas the transglycosylation activities were higher in the straight juices in line with the higher total activity. In general, the higher substrate concentration and the resultant higher viscosity of the concentrated juice samples may limit the mass transfer and availability of nutrients for growth and metabolic activity of the starter organisms, which may result in lower production of fructosyltransferase enzymes. There was no significant difference between the two *L. gasseri* strains in the level of production of fructosyltransferase enzymes in carrot juice.

### 3.2. Sugar Reduction and Change in Total Polysaccharide Content

**Figure 1:**
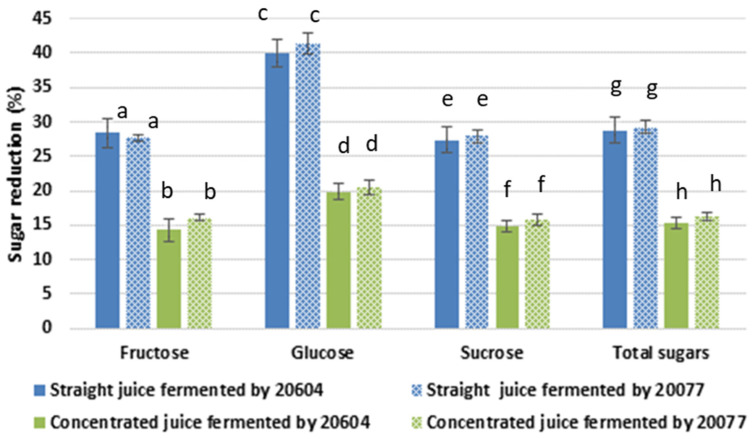
The extent of individual and total sugars reduction (%) in the fermented carrot juices by two *Lactobacillus gasseri* strains (strain DSM 20604 and strain DSM 20077) compared to non-fermented juices. Different letters within a group indicate significant difference (*p* < 0.05).

Fermentation of straight and concentrated carrot juices by *Lactobacillus gasseri* (DSM 20604 and DSM 20077) for 24 h resulted in a significant reduction in the concentration of fructose, glucose, and sucrose (Figure 1). The extent of the individual and total sugar reduction was independent of the starter cultures used in this study. There was a greater sugar reduction (~27.0%) in fermented straight carrot juices than in fermented concentrated juices (~15.8%), which could be due to limitation in growth and metabolic activity in the concentrated juices as mentioned above. The total sugar content of the straight carrot juice was 89 mg/mL, of which ~82% was sucrose. This was reduced to 63.3 and 62.8 mg/mL during fermentation by DSM20604 and DSM20077, respectively. The sugar content of the carrot juice concentrate was on average 136 mg/mL. This was reduced to 115.1 and 113.7 mg/mL respectively in carrot juice concentrates fermented by DSM20604 and DSM20077. The highest level of percentage reduction in both straight and concentrated carrot juices were observed for glucose, whereas similar levels of sugar reduction were observed in the case of fructose and sucrose. However, in terms of absolute amounts metabolised, sucrose was the most utilised (~20 mg/mL), followed by glucose (~3.6 mg/mL) and fructose (~2.1 mg/mL) in the fermented straight juice samples with no significant difference between the two strains. The trend was similar in the juice concentrates fermented by both strains, although the absolute amounts were slightly lower (~17 mg/mL, ~2.7 mg/mL, and ~1.7 mg/mL for sucrose, glucose, and fructose, respectively). The reduction in the level of simple sugars in the fermented samples can be partially explained by the utilisation of the sugars for the growth and metabolic activities of the fermenting organisms. Previous studies also reported total sugar reduction of 10.3% and 19.3% during carrot juice fermentation by *Lactabacillus helveticus* JCM-1120 [30] and by *Lactobacillus rhamnosus GG* [31], respectively.

The observed reduction in sucrose can be attributed to hydrolysis by fructosyltransferase enzymes into fructose and glucose. However, no increase in glucose and fructose was observed after fermentation, indicating that the liberated monosaccharides could be consumed by *Lactobacillus gasseri* strains as their energy sources or utilised in biosynthesis of prebiotic fructo-oligosaccharides and polysaccharides. *Lactobacillus gasseri* DSM20604 is known to produce inulosucrase and levansucrase, which catalyze the hydrolysis of sucrose into fructose and glucose and transglycosylation into fructo-oligosaccharides and inulin and levan, respectively. Similarly, DSM20077 produces levansucrase [18]. As discussed in Section 3.1, significant activities of fructosyltrasferase enzymes were observed in the straight and concentrated carrot juices fermented by both *L. gasseri* strains. Only trace amounts of oligosaccharides were detected in the fermented juices, although earlier studies showed that *L. gasseri* DSM20604 has the capacity to synthesise oligosaccharides with DP 2 to DP 13. The difference in the composition of the substrate, i.e., MRS with sucrose versus carrot juice, may explain the observed difference. On the other hand, a substantial increase in total polysaccharide concentration of the juices ranging from 25 to 77% were observed after fermentation (Table 1), which supports the argument that the reduction in simple sugars was partially due to enzymatic conversion into inulin and levan catalyzed by fructosyltransferase enzymes produced by the starter organisms. There was no substantial decrease in the Brix of the juices after fermentation confirming that most of the soluble solids, i.e., the simple sugars were converted into other soluble metabolites. The °Brix decreased from 8.5 to 8.0 in the straight juice whereas the brix decreased from 15.2 to 14.8 in the juice concentrates after fermentation irrespective of the strain. Interestingly, there was no significant difference (*p* > 0.05) among the various samples in the level of increase in polysaccharide content after fermentation. Although, the total level of sugar reduction was lower in the fermented juice concentrates, the increase in the level of polysaccharides was similar to the single strength juices indicating that a larger proportion of the sugars were utilised for polysaccharide formation in the concentrated juices as opposed to growth and other metabolic activities. Before fermentation, there were 89 mg/ mL simple sugars and 11.8 mg/mL polysaccharides in straight carrot juice. After fermentation of straight carrot juice by *L. gasseri* DSM 20604 and DSM 20077, the total sugar utilisation was 26.2 and 25.5 mg/mL and polysaccharide formation were 9.1 and 2.9 mg/mL, respectively. This implies about 34.9% of the sugar was utilised for conversion into polysaccharides during fermentation by *L. gasseri* DSM 20604 while 11.5% was utilised for conversion into polysaccharides during fermentation by *L. gasseri* DSM 20077. The carrot juice concentrate contained 136 mg/mL simple sugars and 24.7 mg/mL polysaccharides. After fermentation, the concentration of polysaccharides increased to 40.5 and 37.3 mg/mL by *L. gasseri* DSM 20604 and *L. gasseri* DSM 20077, respectively, whereas the total sugar utilisation was 20.7 and 22.1 mg/mL, respectively. Thus, 73.7% of the utilised sugar was converted to polysaccharides during fermentation by *L. gasseri* DSM 20604 and 57.4% during fermentation by *L. gasseri* DSM 20077 of the concentrated carrot juice.

### 3.3. Change in Titratable Acidity of Carrot during Fermentation

**Figure 2:**
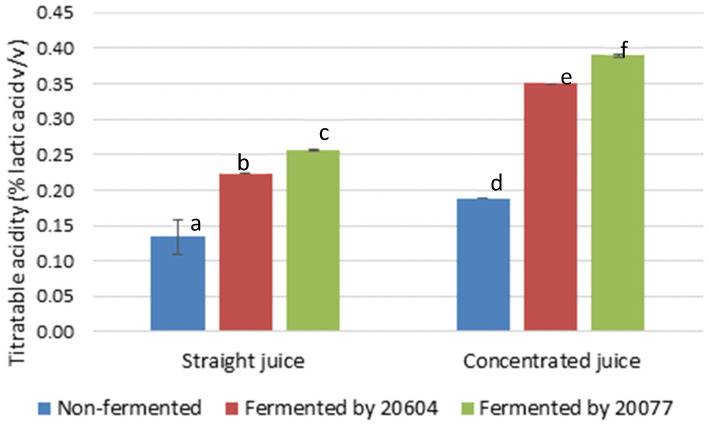
The titratable acidity in straight and concentrated juices before and after fermentation by *L. gasseri* strains. Different letters within a group represent significant difference (*p* < 0.05).

Titratable acidity is an important quality attribute, which determines the sensory quality and acceptability of beverages. During fermentation of straight juices with *L. gasseri* DSM 20604 and DSM 20077 the titratable acidity increased by 1.7-fold and 1.9-fold (Figure 2). The highest titratable acidity (0.39% lactic acid equivalents) was recorded in concentrated carrot juice fermented by *L. gasseri* DSM 20077 corresponding to a 2.1-fold increase compared to initial acidity. In earlier studies [30,32], the titratable acidity of carrot juices fermented with different *Lactobacillus* strains varied from 0.25 to 0.54%. It has to be noted that titratable acidity is a gross measure of the titratable acids present in a product and is dependent on the type and concentration of acids originally present in the raw material, formation of organic acids during fermentation as well as fermentation induced interconversion of organic acids. For instance, malic acid is a dicarboxylic acid contributing twice as much to titratable acidity compared to lactic acid which is a monocarboxylic acid. The amount of lactic acid produced by fermentation depends on type of lactic acid bacterial used, the amount of available sugar present in the substrate and on other substances present or added into the substrate which support or suppress lactic acid production [5]. It has been reported that malic acid which is present in small concentration in carrot juice can be converted to lactic acid by decarboxylase reaction during fermentation by some *Lactobacillus* strains [30], contributing to the overall change in titratable acidity during fermentation. However, no reports describing the malolactic activity of *Lactobacillus gasseri* strains were found in the literature.

The increasing trend in titratable acidity was accompanied by a decrease in pH, that was, from pH ~6.0 in non-fermented juices to pH 5.0 and 4.9, and pH 5.1 and 5.0 in fermented straight and concentrated juices, fermented by DSM20604 and DSM20077, respectively. Clearly, the final pH of these products is not sufficient to guarantee the safety and stability of the final product during storage and thus, further fermentation process optimisation and/or acidification to pH < 4.5 is required to maintain product safety and stability.

### 3.4. Polysaccharide, Carotenoid, and Carotene Content Analysed from Raman and FTIR Intensity

**Figure 3:**
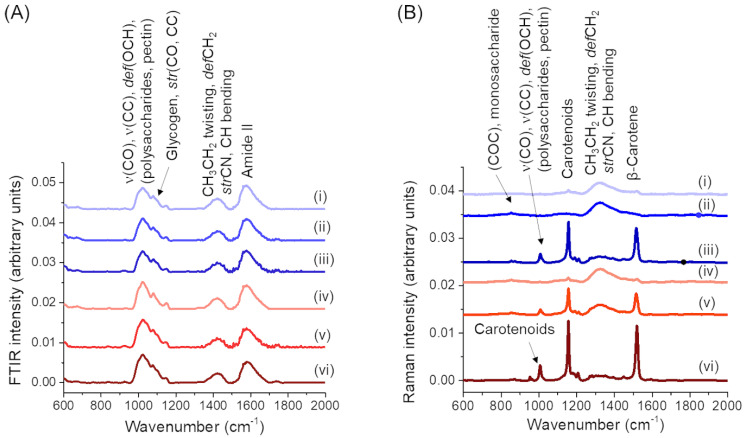
Averaged, intensity-normalised and background subtracted (**A**) FTIR and (**B**) Raman spectra of unfermented carrot juices and fermented carrot juices by *L. gasseri* strains: (i) Unfermented straight juice, (ii) Straight juice fermented by DSM 20604, (iii) Straight juice fermented by DSM 20077, (iv) Unfermented concentrated juice, (v) Concentrated juice fermented by DSM 20604, (vi) Concentrated juice fermented by DSM 20077. Abbreviations: *def*, deformation; *str*, stretching; *υ*, vibration. Assignments are based on studies in the references (Table 2).

The FTIR and Raman spectra comparing fermented carrot juice samples with unfermented juice samples are shown in Figure 3. The most dominant peaks observed in all FTIR and Raman spectra are associated with polysaccharides, protein, and carotenoids (peaks assignments shown in Table 2 are based on studies in the references). When comparing the FTIR spectra (Figure 3A), the intensity of the peaks related with polysaccharides and pectin were observed to be higher in the concentrated juice samples than the straight juice samples. However, there was no obvious and/or little peak alterations were noticed between the spectra of unfermented and fermented samples.

In contrast, noticeable changes in the Raman peaks related with polysaccharides, pectin, carotenoids, and β-Carotene were observed between unfermented and fermented samples of both strains, perhaps due to the higher sensitivity of Raman spectroscopy. The glucose-saccharide peak or (C-O-C) skeletal mode of monosaccharides (α-glucose) and disaccharide (maltose) at the wavenumber around 840–847 cm^−1^ was noticeable in the Raman spectra of all fermented and unfermented samples, while the intensity of this peak became more noticeable after fermentation. The carotenoids related peak at the wavenumber 954–956 cm^−1^ was found only in the DSM20077 fermented concentrated juice. The peak related to vibrational mode of polysaccharides and pectin (υ(CO), υ(CC), δ(OCH)) at the wavenumber 1008 cm^−1^ was observed in DSM20604 fermented concentrated juice and DSM20077 fermented straight and concentrated juice samples. The two prominent peaks attributed to carotenoids i.e., peaks due to C-C and conjugated C=C stretching (in the region of 11,501,157 cm^−1^), and β-carotene peaks from C-C stretching (in the region of 1517–1520 cm^−1^) are noticed in all carrot juice samples as would be expected. Compared to unfermented juices, these peaks were more intense in all fermented juices except for the fermented straight juice by strain DSM20604.

The spectral region related to the C−H vibrations (in the range of 1313–1491), was investigated to identify differences in lipid or protein composition between samples (Figure 3B). The twisting and deformation mode in chemical structure of lipids could be observed in all unfermented and fermented juice samples at the wavenumbers of 1314 cm^−1^ (CH_3_CH_2_ twisting mode of lipid) and 1437–1453 cm^−1^ (deformation of CH_2_). The peak related to C-N stretching vibration coupled with the in-plane C-H bending in amino radical cations at the wavenumber 1491 cm^−1^ is also observed in this region.

**Figure 4:**
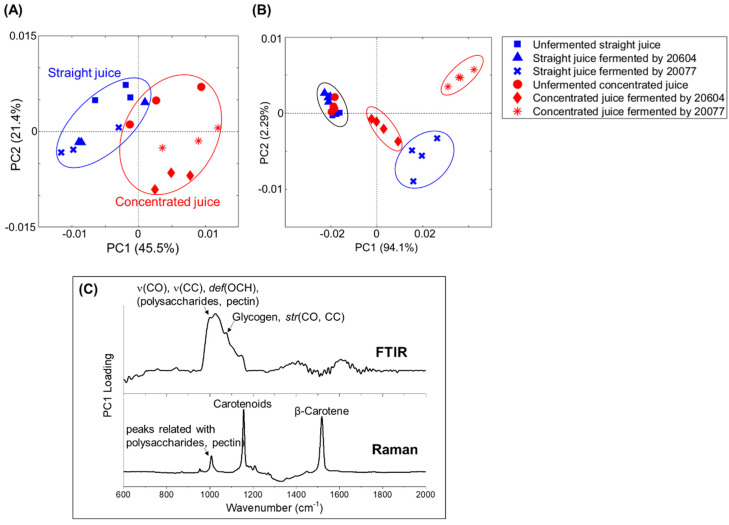
Scatter plots of first and second principal components (PC1 and PC2) from PCA of (**A**) FTIR spectra, (**B**) Raman spectra taken from unfermented carrot juices and fermented carrot juices by two *L. gasseri* strains and (**C**) loading values plot for PC1 from PCA.

PCA was performed on all FTIR and Raman spectra to extract the relevant chemical information related with the spectral alterations observed from modification in these cellular components among control unfermented and fermented carrot juices (Figure 4). The scores plot from PCA of FTIR (Figure 4A) shows a distinct grouping between straight and concentrated juice samples although there were overlapping within the straight juice samples. The first principal components (PC1) was sufficient to differentiate the concentrated juice fermented by both strains from all other samples which accounted for over 40% of the variance in the data.

From PCA of Raman spectra (Figure 4B), a good clustering of each samples group was seen while there was an overlapping between unfermented juice samples and fermented straight juice by 20604 strain. PC1 was sufficient to differentiate the concentrated juice fermented by both strains and straight juice fermented by 20077 strain from the unfermented samples which accounted for over 93% of the variance. From the scores plots, there was (1) a clear separation between unfermented (straight or concentrated) juice and fermented juice by DSM 20077; (2) a clear separation between unfermented concentrated juice and fermented concentrated juice by DSM 20604, while there was no big difference between unfermented straight juice and fermented straight juice by DSM 20604; (3) the juice concentration affects the macromolecular changes during fermentation. The dominant spectral variation observed in the PC1 loading plots of FTIR and Raman spectra (Figure 4C) confirm that the main difference between these samples were associated with polysaccharides and pectin (υ(CO), υ(CC), δ(OCH)), lipid related peaks (CH_3_CH_2_ twisting and deformation), carotenoids, and β-carotene.

**Figure 5:**
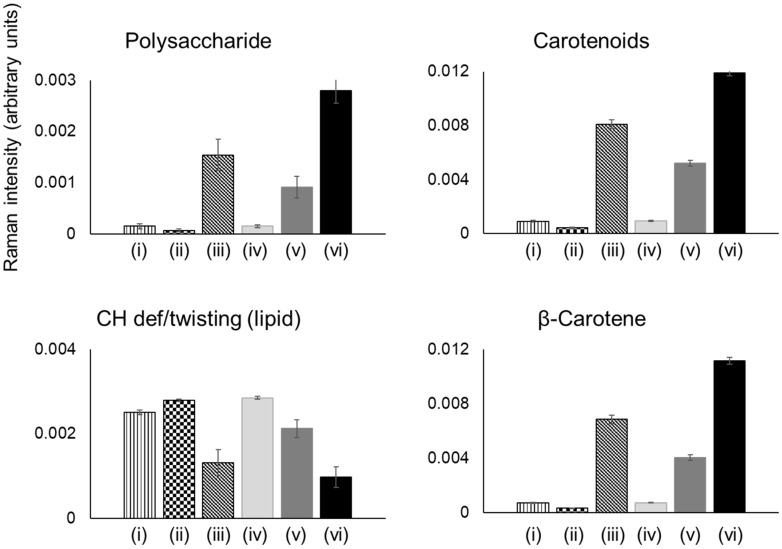
Intensity changes of polysaccharide, carotenoids, lipids, and β-carotene specific peaks in the Raman spectra of fermented single strength/concentrated juices (fermented by *L. gasseri* strains) comparing to unfermented juices: (i) Unfermented single strength juice, (ii) Single strength juice fermented by DSM 20604, (iii) Single strength juice fermented by DSM 20077, (iv) Unfermented concentrated juice, (v) Concentrated juice fermented by DSM 20604, and (vi) Concentrated juice fermented by DSM 20077.

Specific peak analysis of the Raman spectra was further performed to compare those changes in fermented juice samples with unfermented samples (Figure 5). The prominent peaks observed in the loadings plot were selected for the analysis. Compared to unfermented juices, increased peak intensities associated with polysaccharide, carotenoids, and β-carotene were observed in all fermented juices by both strains except the single-strength juice fermented by DSM 20604. The increase was observed to be higher in all juices fermented by strain DSM 20077 compared to the fermented juices by strain DSM 20604. The observed increase in carotenoides content of carrot juice after fermentation are comparable with the results previously reported for carrot juice fermented by *L. plantarum* where an increase in β-carotene content was observed after fermentation [38]. Moreover, Chavasit et al. [39] reported that β-carotene contents were increased during fermentation of pickled vegetables. In our study, the increase in carotenoid content was observed in the concentrated juice fermented by DSM 20604 and in both straight and concentrated juices fermented by DSM 20077. The observed increase during fermentation could be explained by two factors (1) the synthesis of carotenoids by *L. gasseri* during the fermentation process and/or (2) increased release and detection of carotenoids due to macromolecular changes in the juice during fermentation. Lactic acid bacteria are known to synthesise carotenoids as a protective mechanism against oxidative stress [40], although to the best of our knowledge, there are no reports in literature on synthesis of carotenoids by *Lactobacillus gasseri*. It is possible that *L. gasseri* experiences a higher level of stress in the concentrated juice samples, which may explain the observed higher intensity of carotenoid peaks in the concentrated juice samples after fermentation by both strains. Lactic acid bacteria fermentation can also enhance the release and bioaccessibility of phytochemicals such as carotenoids in plant matrix. Lactic acid fermentation of tomato pulp with different lactic acid bacteria resulted in 33.6 to 41.1% increase in total carotenoids, 24.8 to 50% increase in lycopene and up to 69% increase in β-carotene content [41].

The Raman peak analyses indicate that fermentation by strain DSM 20077 results in higher level of polysaccharides and carotenoids during fermentation of both straight and concentrated juices compared to strain DSM 20604. In addition, fermentation of concentrated juice results in more polysaccharide formation compared to straight juice. On the other hand, based on the total polysaccharide analysis, there was no significant difference in relative production of polysaccharides by both strains during fermentation of straight and concentrated juices. Perhaps, only a portion of the polysaccharides in the samples were detectable by Raman analysis. Further analysis of the samples for specific polysaccharides and carotenoids is required in order to better understand the impact of fermentation by the two strains on the polysaccharide and carotenoid contents of carrot juices of different concentrations.

## 4. Conclusions

This study showed that fermentation by *Lactobacillus gasseri* DSM 20604 and DSM 20077 enables the manufacture of functional carrot juices enriched with prebiotic polysaccharides and carotenoids. Raman spectroscopic analysis indicated that DSM 20077 results in higher carotenoid concentration and polysaccharide formation during fermentation of specially concentrated carrot juice. Nevertheless, further studies are required to characterise and quantify the polysaccharides and carotenoids formed during the process.

## Figures and Tables

**Table 1:** The activity of fructosyltransferase enzymes and relative changes in total polysaccharide contents (the ratio of fermented to non-fermented samples) in carrot juice samples fermented by *L. gasseri* DSM 20604 and DSM20077. Similar letters in the prefixes within column indicate no significant difference (*p* > 0.05). The estimated crude polysaccharide contents of the straight and concentrated carrot juices were 11.7 mg/mL and 24.7 mg/mL, respectively.

Sample	Enzyme Activity (U/L Juice)			Relative Change in Total Polysaccharide Content
	Total Activity	Hydrolytic Activity	Transglycosylation Activity	
Fermented straight DSM 20604	570 ± 115.6 ^a^	290 ± 59.3 ^b^	280.9	1.77 ± 0.17 ^c^
Fermented straight DSM 20077	530 ± 51.9 ^a^	290 ± 32.3 ^b^	240.7	1.25 ± 0.22 ^c^
Fermented concentrated DSM 20604	370 ± 171.5 ^a^	250 ± 10.6 ^b^	113.5	1.64 ± 0.04 ^c^
Fermented concentrated DSM 20077	350 ± 44.2 ^a^	290 ± 62.1 ^b^	61.2	1.51 ± 0.27 ^c^

**Table 2:** Selected Fourier Transform Infrared (FTIR) and Raman frequencies and their peak assignments for the spectra shown in Figure 3 and Figure 4**.**

Wave Number (cm^−1^)	Peak Assignment
FTIR spectra	
963–1018	υ(CO), υ(CC), *def*(OCH), ring (polysaccharides, pectin)
1020–1050	Glycogen absorption due to *str*(C-O and C-C) and *def*(C-O-H)
1400–1500	Symmetric CH_3_ bending of the methyl groups of proteins, *str*(C-N), *def*(N-H), *def*(C-H)
1480–1543	Amide II
Raman spectra	
954–956	Carotenoids
1008	υ(CO), υ(CC), *def*(OCH), ring (polysaccharides, pectin)
1150–1157	Carotenoid peaks due to C-C and conjugated C=C band stretch, C-C, C-N stretching (protein),
1313–1314	CH_3_CH_2_ twisting mode of collagen/lipid
1325–1339	CH_3_CH_2_ wagging mode in purine bases of nucleic acids and tryptophan
1370	The most pronounced saccharide band
1437–1453	*def*CH_2_
1462	*def*CH_2_ of disaccharides, sucrose
1491	C-N stretching vibration coupled with the in-plane C-H bending in amino radical cations
1517–1520	β-carotene accumulation (C-C stretch mode)
1520–1538	Carotenoid peaks due to C-C and conjugated C=C band stretch

Abbreviations: *def*, deformation; *str*, stretching; *υ*, vibration. Peak assignments are based on earlier Raman [33,34,35] and FTIR [36,37] studies.
